# How did South Africans with disabilities experience COVID-19? Results of an online survey

**DOI:** 10.4102/ajod.v12i0.1119

**Published:** 2023-02-09

**Authors:** Mary Wickenden, Tim G.B. Hart, Stephen J. Thompson, Yul D. Davids, Mercy Ngungu

**Affiliations:** 1 Participation, Inclusion and Social Change Cluster, Institute of Development Studies, Brighton, United Kingdom; 2 Developmental, Capable and Ethical State Research Division, Human Sciences Research Council, Pretoria, South Africa; 3 Department of Sociology and Social Anthropology, Faculty of Humanities, Stellenbosch University, Stellenbosch, South Africa; 4 Developmental, Capable and Ethical State Research Division, Human Sciences Research Council, Cape Town, South Africa; 5 Impact Centre Research Division, Human Sciences Research Council, Pretoria, South Africa

**Keywords:** South Africa, disability, COVID-19, rights, socio-economic, survey, online

## Abstract

**Background:**

People with disabilities are a large, disadvantaged minority, comprising approximately 12% of the population. The South African government has ratified international and regional disability treaties but deals with disability rights within general anti-discrimination legislation. There are no specific frameworks to monitor justice for people with disabilities. The study aims to inform further development of disability inclusive mechanisms relating to crises including pandemics.

**Objectives:**

This study explored the perceptions of South Africans with disabilities, to understand their experiences during coronavirus disease 2019 (COVID-19), focussing on socioeconomic, well-being and human rights aspects.

**Method:**

An online survey tool generated quantitative and qualitative data. Widespread publicity and broad recruitment were achieved through project partners networks. Participants responded via mobile phone and/or online platforms.

**Results:**

Nearly 2000 people responded, representing different genders, impairments, races, socio-economic status, education and ages. Findings include: (1) negative economic and emotional impacts, (2) a lack of inclusive and accessible information, (3) reduced access to services, (4) uncertainty about government and non-government agencies’ support and (5)exacerbation of pre-existing disadvantages. These findings echo international predictions of COVID-19 disproportionally impacting people with disabilities.

**Conclusion:**

The evidence reveals that people with disabilities in South Africa experienced many negative impacts of the pandemic. Strategies to control the virus largely ignored attending to human rights and socioeconomic well-being of this marginalised group.

**Contribution:**

The evidence will inform the development of the national monitoring framework, recognised by the South African Government and emphasised by the United Nations as necessary to ensure the realisation of the rights of people with disabilities during future crises including pandemics.

## Introduction

The first global case of coronavirus disease 2019 (COVID-19) was recorded in late 2019 and by March 2020 the World Health Organization (WHO) confirmed that the world was facing a pandemic (WHO 2020b). In pre-pandemic times, people with disabilities were recognised as one of the most excluded groups in our society, despite the United Nations (UN) Convention on the Rights of Persons with Disabilities (CRPD) declaring that people with disabilities cannot be discriminated against and should enjoy all human rights and fundamental freedoms (UN 2007). As the seriousness of the pandemic developed, warnings emerged that COVID-19 could deepen pre-existing inequalities for people with disabilities, exacerbating their experiences of social and economic exclusion (UN 2020). South Africa is a signatory to the CRPD and the Optional Protocol and has ratified both.

As the situation evolved, the UN (2020) urged states to honour their international commitments and include people with disabilities in COVID-19 responses. Meanwhile, the first indications from South Africa were emerging that this was not happening and people with disabilities were being excluded (International Disability Alliance [IDA] 2020b; McKinney, McKinney & Swartz 2020). Despite many social researchers around the world pivoting their work to focus on better understanding the human impact of the pandemic and associated responses, a paucity of evidence relating to how marginalised groups were being included or excluded from research remained.

While some studies (such as Stiegler & Bouchard 2020) investigated the social impact of COVID-19 in South Africa, the empirical evidence relating to experiences of people with disabilities remained limited. Part of the challenge is that people with disabilities are often excluded from research. When deciding about whom or what to investigate, researchers often have a bias towards convenient questions and populations that are readily researchable, as opposed to inconvenient questions and populations who are difficult to research (Chambers 2017). As such, not all segments of society are equally included in research. Evidence suggests that COVID-19 research is not uniform, with some societal groups being included while others were excluded. Strachan (2021:3) reported that ‘There is significant coverage of the gendered impact of COVID-19 on research methods and approaches’, but an evidence gap exists relating to how people with disabilities experienced the pandemic and national responses.

To address this evidence gap, this research explores the impact of COVID-19 on people with disabilities in South Africa. To investigate the social and economic impact and human rights aspects of the pandemic on this marginalised and often under-researched group, a research partnership was formed between two South African partners, the Human Sciences Research Council (HSRC) and the National Council of and for Persons with Disabilities (NCPDs), and the Institute of Development Studies (IDS) from the United Kingdom. The objective was to produce evidence-informed recommendations that the South African government and civil society can use to help to ensure that the rights of South Africans with disabilities are upheld when executing measures to diminish against the impact of the current pandemic and future crises.

### Background literature

A review of literature on the impacts of COVID-19 on people with disabilities globally finds that there is a limited but growing body of work. There is a particular dearth of evidence focusing specifically on Africa. The current evidence tends either to be focused on a few countries or form part of large global surveys, but these often have very few respondents from middle- and low-income countries. Several extensive literature reviews have been undertaken (Kubenz & Kiwan 2021; Meaney-Davis, Lee & Corby 2020; Wickenden et al. 2022).

Much of the global level grey literature, published early in the pandemic, subsequently predicted exacerbated negative experiences for people with disabilities (Castres & O’Reilly 2020). Advisory materials written by Organisations for People with Disabilities (OPDs), International Non-Governmental Organisations (INGOs) and various UN bodies, although variously focussed on specific subgroups of people with disabilities or allied workers, are generally in agreement about calling for a universally disability inclusive approach to humanitarian action and pandemic mitigation (CBR Africa Network [CAN] 2020; Hartley & Balakrishna 2020; HI 2020; IDA 2020a; International Disability Alliance & International Disability and Development Consortium [IDA & IDDC] 2020; Leonard Cheshire 2020; Light for the World 2020; Office of the United Nations High Commissioner for Human Rights [OHCHR] 2020; UNCRPD 2020; United Nations Educational, Scientific and Cultural Organization and United Nations Partnership on the Rights of Persons with Disabilities [UNESCO and UNPRPD] 2021; WHO 2020a; World Blind Union [WBU] 2020).

Some research has explored the impacts and challenges imposed by the pandemic with groups who are allies of people with disabilities such as OPDs and agencies such as INGOs whose activities have been affected by COVID-19 (Bhakta 2021). The limited primary research on COVID-19 and disability, asking people with disabilities directly about their experiences, is mostly focussed on high income settings, on populations with particular health concerns, genders or impairments (Smith et al. 2020; Women Enabled International [WEI] 2020).

By nature, the pandemic and the restrictions on personal contact made it difficult to conduct any kind of face-to-face research, such as interviews or focus groups, and so many have used online methods, either structured, semi-structured or narrative-based methods (i2i 2020; Rohwerder et al. 2021). Some have explored aspects such as health or economic concerns or access to support and relief services, but often those from the most marginalised impairment groups have been left out, because of the perceived challenges in accessing their views (Meaney-Davis 2020). Fewer have asked broad questions related to socio-economic impacts, emotional well-being or human rights related aspects or explored the effects of the different types of lockdowns (i.e. different levels of strictness), which many countries’ governments imposed at different times. Issues related to access to vaccinations and prioritisation for healthcare have been explored to some extent, but came later in the progress of the pandemic, as vaccines, protocols and clearer ideas about treatment developed (Epstein, Ayers & Swenor 2021; McKinney et al. 2020; McKinney, McKinney & Swartz 2021; WHO and UNICEF 2021). The present authors have reported on some of our data on vaccination separately (Hart et al. 2021).

Overall, findings from studies looking at impacts of the pandemic on individuals with impairments have been remarkably (and sadly) uniform across countries and regions, subgroups explored, and methodologies used. Thus, individual interviews (usually conducted remotely by phone), online surveys, group responses from representatives such as OPDs, all report the exacerbated disadvantages experienced by many people with disabilities globally during the pandemic (Bernard et al. 2020; Brennan et al. 2020). Economists are now exploring the extent to which the pandemic has increased inequity and it seems clear that it has made the already poor, poorer in many situations (Pozhidaev 2022).

At a more granular level, there are examples in diverse settings of the emotional and psychological impact – including stress and/or depression and/or worry about immediate survival and the future, feelings of loss (of opportunities, and of agency), increased conflict within the home and outside, and feeling isolated and confined. Economic impacts are also widely reported, where people have lost work or their own businesses and some have had extra expenses, such as increased costs of transport, food, impairment related services (Christensen 2020; Rohwerder et al. 2021).

Often there was worry about how they would cope financially if there were repeated waves of lockdown, as savings and loan options had been exhausted early on and many had serious concerns about falling into dire poverty.

Educational opportunities were limited, as schools, colleges and universities were closed and, of course, this affected all learners everywhere. However, for students with disabilities, who anyway are likely to live in poverty, access to online learning was often reduced because of the lack of connectivity or because virtual learning was not adapted and accessible for them (Ressa 2020). In addition, for some young people with learning disabilities and/or social and/or behavioural difficulties like autism, not being able to go to their regular daytime activities was difficult to understand and caused frustration and challenges for their families at home (Samboma 2021). Social contact with others was missed and for some friendships at home and in the community were more limited, so people felt isolated and lonely (Courtenay & Cooper 2021; Rohwerder 2020).

Another major area of consensus, across many studies and settings is the general inadequacy of information from government and other agencies in accessible formats (Armitage & Nellums 2020; Fernandez-Diaz, Iglesias-Sanchez & Jambrino-Maldonado 2020; Yap et al. 2020). Television announcements are often not sign language or caption supported, visual or written information on posters and leaflets may not be accessible, content is often not in easy read, simple language with visual support, and so on. Various studies found that people with disabilities felt they did not have access to all the accurate information they needed or that messages were confusing.

Relief and support services are also often reported to be insufficiently inclusive and adaptive. For example, having to go to a central collection point to acquire hygiene products, food, and the like is not easy. There is report of helpful support (both emotional and material) from OPDs and INGOs, but this was not enough. Commonly people felt that the government’s emergency relief processes had not taken the needs of people with disabilities into account. This included confusion and ambiguity about social protection and the relationship between ordinary (pre-COVID-19) disability grants and similar, and what was available during COVID-19 (Hart, Msitshana & Bohler Muller 2020).

Gender, age and impairment specific concerns arise to some extent in the literature (Wickenden et al. 2022). For example, women and girls sometimes report increased violence or perceived risk of violence both at home and in public spaces. Both children and the elderly are mentioned as vulnerable and like people with disabilities across the lifespan could not always access their usual health and rehabilitation services and community supports. Fear of infection and increased stigma and discrimination against people with disabilities from the community were evidenced in some studies (McKinney et al. 2021; Ned et al. 2020).

Some authors interestingly discuss the more existential aspects of disability status and whether vulnerability is a useful or acceptable term to use especially in the context of an extreme situation such as a pandemic. It is certainly true that some people with disabilities feel patronised and disempowered by being described as vulnerable, while others argue that this status is important in achieving extra recognition and protection (such as priority access to health services, vaccines etc.) (Ahmad et al. 2020; Ned et al. 2020; Rotarou et al. 2021; Scully 2020; Singh 2020).

There were some recurring impairment-related challenges; for example, for people with mobility impairments – difficulty in accessing emergency support venues; for visually impaired people – concerns about the safety of accepting physical contact for guiding as they feared infection through direct contact; for hearing impaired people – misunderstandings about or the lack of access to the key information; for those with psychosocial impairments – the potential lack of access to their usual medication and psychological support and exacerbation of mental health difficulties such as depression and anxiety (Sale, Polyakov & Eaton 2020).

To summarise, negative impacts of COVID-19 for people with disabilities were presaged early in the pandemic by both researchers and people with disabilities and their allies in OPDs and by UN agencies and INGOs and have generally been found to be true predictions.

Although South Africa still lacks specific disability legislation in the form of an *Act of Parliament, section 7(2) of the 1996* Constitution enjoins the State to ‘respect, protect, promote and fulfil the rights in the Bill of Rights’. It enshrines the rights of all who reside in South Africa, including people with disabilities, to equal treatment covering various aspects including their rights to dignity, personal security, freedom from all types of violence, access to adequate housing, healthcare services, sufficient food and water, social security, basic education, and adult basic education. Section 9 of the Constitution, in the Bill of Rights, guarantees equality and prohibits discrimination for everyone, including people with disabilities. The *Promotion of Equality and Prevention of Unfair Discrimination Act 4* (2000) and the *Employment Equity Act 55* (1998) jointly give effect to the implementation of section 9.

During the early stages of the pandemic, McKinney et al. (2021) identified that despite these legal protections people with disabilities were facing continuing challenges regarding rights to accessing healthcare and related services. Interestingly, neither the *Disaster Management Act of 2002* and the Disaster Management Framework (Republic of South Africa, 2005), the legislation and policy invoked since March 2020 to manage and mitigate the COVID-19 pandemic, make any mention about people with disabilities. Instead they talk of vulnerable groups and households. Consequently, they ignore the diversity of vulnerability of people with disabilities and thus would seemingly fail to adequately acknowledge this group and ensure that interventions focus on their specific circumstances and needs.

This study set out to understand the experiences of people with disabilities in South Africa, within the context of the country’s specific regulations and various levels of lockdown. The authors focussed particularly on socioeconomic well-being and human rights related aspects and with the intention to inform the government frameworks and practice in relation to disability inclusive disaster and crises management.

## Research methods and design

The researchers’ approach was to design a bespoke survey to be disseminated online as widely as possible to people with disabilities across South Africa. Participants responded via phones and computers and their data were uploaded and stored centrally and anonymously. To ensure that potential participants did not refrain from joining the study for financial reasons, respondents were compensated for the data costs incurred through their participation.

The research team comprised members from the three collaborating partner institutions. The team jointly compiled and tested an online survey specifically for people with disabilities exploring a wide range of socioeconomic issues related to COVID-19 and with accessibility needs particularly considered. The draft survey was piloted among a group of people with diverse disabilities and disability scholars. After adjustment, it was made available online as a Google Forms document.

To comply with COVID-19 protocols that prevented direct access to individuals with disabilities, a covering letter with the link to the survey was sent to many people with disabilities, OPDs, government departments and private enterprises, particularly those known to support and employ people with disabilities, asking them to disseminate it to persons with disabilities. The introduction included a consent process and guidelines for parents or assistants who could support respondents where necessary. These procedures were in accordance with both the IDS and the HSRC research ethics approval obtained for this study. The online survey introduction asked respondents to complete the questionnaire if they considered themselves a person with disabilities or with at least one impairment. The research team had no direct contact with the respondents and only received anonymous data from the online data set of voluntary respondents. A total of 1857 respondents completed the survey.

The survey tool was quantitative in structure but included some free-text questions to elucidate some of the quantitative answers in more detail. The instrument focused on general socioeconomic and human rights experiences of persons with disabilities. It collected some demographic information including the Washington Group (2019) short set of questions. The main questions asked were about economic and emotional status and impacts, perceptions of support by the state and other organisations during the pandemic, communication and awareness of COVID-19, access to usually needed care services, willingness to be vaccinated, and perceived risk of and exposure to COVID-19. Also included were questions about perceptions of the disability-inclusiveness of government and non-governmental organisations (NGO) responses to COVID-19. This analysis uses descriptive statistics and focuses on the quantitative questions. However, some qualitative clarification responses were invited where relevant. The authors used SPSS version 27 to analyse the quantitative data. Data were disaggregated and cross-tabulated in selected ways for reporting in this article.

### Ethical considerations

The authors gained ethical approval for the study from both IDS (UK) ethics committee and Human Science Research Council (SA) Ethics Committee protocol No. REC 1/11/20.

## Results

### The sample

A detailed analysis of the 1857 respondents showed that 63.9% (*n* = 1185) were male, 35.5% (*n* = 660) were female, and 0.6% (*n* = 11) indicated other. In terms of education, the majority completed some secondary schooling (22%, *n* = 406); some completed matric (40%, *n* = 745) and higher education or a degree or diploma (16%, *n* = 294). Twelve per cent had no formal schooling (*n* = 219) and 4% only had primary schooling (*n* = 81). A combined total of 6% selected ‘do not know’ or ‘prefer not to say’ regarding the questions on education level. A large proportion of the sample (93%; *n* = 1736) is 18–54 years of age. Almost one-third (31%, *n* = 579) were between 18 and 24 years; 21% (*n* = 395) 25–34 years; 13% (*n* = 234) 35–44 years; and 28% (*n* = 528) 45–54 years. South Africa is a highly unequal country, because of its apartheid legacy of racial inequalities, thus the authors provide a disaggregation by race using the usual categorisations, although they recognise that these are potentially problematic. The survey sample was largely black African people (83%, *n* = 1544), while white people (10%, *n* = 186), mixed race people (5%, *n* = 93), and Indian people (2%, *n* = 29) respondents are fewer. The disaggregated figures of the sample, using racial categories, approximately coincide with the racial proportions in the country suggesting that our recruitment strategy was broadly successful in reaching many parts of the population.

**FIGURE 1:**
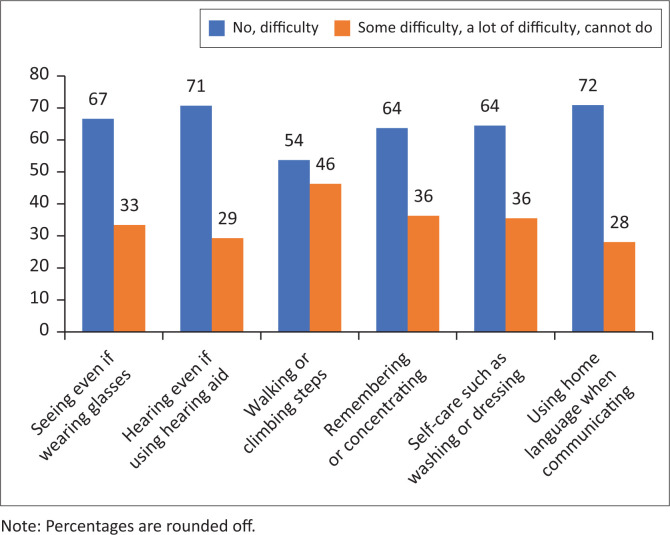
Washington group short set questions: Numbers indicating difficulties within six domains (*N* = 1857).

In relation to impairment, respondents were asked about the functional difficulties they have because of a health problem, using the Washington group short set of questions. Overall, it was found that of the 1857, approximately 71% (*n* = 1330) of participants had more than one functional difficulty. Further analysis reveals that a large proportion of the sample indicated having difficulty (some difficulty, a lot of difficulty or cannot do) with walking and climbing steps (46%, *n* = 860), 36%(*n* = 675) reported difficulty remembering or concentrating, 36% (*n* = 659) had difficulty with self-care, such as washing or dressing and 33% (*n* = 621) had difficulty with seeing even when wearing glasses. Fewer respondents indicated difficulty with hearing even when using a hearing aid or similar assistive device (29%, *n* = 544). Others reported communication difficulties with regard to being understood even when using their home language (28%, *n* = 541) (See Figure 1).

### Thematic results

The data are presented in relation to the key themes that were explored in the survey.

### Economic impacts

When asked about the single worst thing about lockdown, the most commonly given answer was the economic situation, reported by 43% of participants. This was more than double the next most commonly reported answer, which was the lockdown restrictions on movement (20%). Some of the questions asked the participants directly about the impact of the pandemic on their economic situation. Other questions asked indirect questions on the same theme – for example about the impact on accessing food. In general, the data shows that, as might be expected, the pandemic and lockdowns negatively affected the financial situation of many people with disabilities. Three quarters (76%) reported that because of their financial situation, as a result of lockdown measures, they now have difficulties ‘paying for my living expenses’. Only 6% of participants did not agree with this statement. The remaining participants either do not know (4%) or neither agreed nor disagreed (14%).

When asked specifically about their disability-related expenses, 49% felt that their financial situation was now worse than before COVID-19 as income was less or disability-related expenditure had increased. Around 44% felt that their situation was the same, but only 4% said their situation had improved. The remaining 3% responded that they did not know. However, looking ahead, most participants were optimistic, as 51% did not envisage their financial situation worsening in the next few months. A smaller proportion (39%) were concerned that their financial situation was likely to become worse, while 10% responded that they did not know what the future would hold.

The respondents reported their employment status at the start of lockdown on 27 March 2022, 37% (*n* = 688) being in some form of employment, including self-employment, while 35% (*n* = 650) indicated being unemployed. Around 11% were physically unable to work, while others were pensioners and students or were doing unpaid caring work. With regard to how the pandemic affected the employment status of participants, responses to a multiple response question indicated that 13% (*n* = 241) of the total sample (*n* = 1857) reported being made redundant because of lockdown measures. A further 11% (*n* = 204) of all participants reported having had their income reduced – meaning they were doing the same work for less money. As a result of the nature of multiple response questions, there may be overlaps in the responses, suggesting that those who were working for less money may have also experienced losing their jobs eventually. Similarly, those who lost their jobs may have obtained jobs later for lower pay. The impact on employment (losing employment or being paid less) made running out of money a reality for some participants. The data suggest that this economic impact resulted in many participants also facing food insecurity and may have also affected the ability to secure products or services.

When asked about events that were not normally experienced but had been during the pandemic, 39% of participants reported that they had gone to bed feeling hungry, and the same number reported someone in their house having gone to bed hungry. Forty per cent said they had run out of money to buy food. Shockingly, at the time of the survey, and despite 15 months of government and NGO interventions, 29% of the respondents reported that they or somebody else in their household had gone to bed hungry during the previous seven days prior to completing the survey.

Interrupted access to electricity at home (42%) was reported as an unusual event by 42%, despite South Africa’s long energy crisis. Running out of soap or sanitiser were also reported as unusual events by 36% of the respondents. However, from the data it is unclear if the access to these products had become worse because of personal economic challenges stemming from the pandemic or wider challenges such as the supply of products that became scarce during the pandemic. Some respondents observed that they needed soap and sanitiser because of specific disabilities and others found that the lack of electricity prevented them from charging assistive devices that subsequently isolated them socially (a lack of mobility and inability to hear).

**FIGURE 2:**
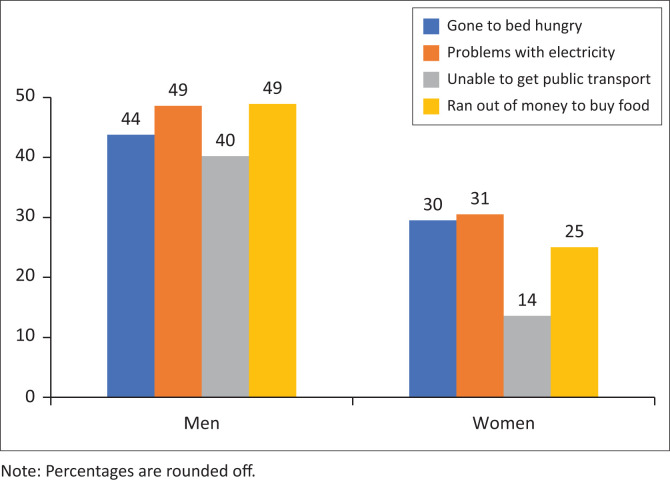
The coronavirus disease 2019 economic impact of lockdown (*N* = 1857).

When these multiple response questions (respondents could select more than one answer and thus the total number of responses does not coincide with the total sample size) are disaggregated by gender, a larger proportion of men (44%, *n* = 519) compared with women (30%, *n* = 195) reported that they went to bed feeling hungry (Figure 2). Men (49%, *n* = 576) are more likely to experience problems with electricity at home than women (31%, *n* = 201), and men (40%, *n* = 477) compared with women (14%, *n* = 90) were less able to get public transport when needed. A larger proportion of men (49%, *n* = 580) than women (25%, *n* = 165) ran out of money to buy food. A disaggregation by race indicated that a larger proportion of black African people (44%, *n* = 683) compared with mixed race (18%, *n* = 17), Indian people or Asian people (10%, *n* = 3) and white people (7%, *n* = 12) have gone to bed feeling hungry.

### Perceptions of services and support

Respondents were asked if they were able to receive all necessary disability-related support during the pandemic, and 45% said they did, while 42% said that they did not and 13% were uncertain. Possible reasons for uncertainty included reliance on others to acquire the necessary supplies and services for them, and respondents’ unawareness of whether these people were struggling to get necessary supplies and services. Under half (45%) reported that the pandemic and subsequent regulations disrupted their disability- or impairment-specific services, including access to medication and psychological or physical rehabilitative therapy.

Approximately 40% reported no interruption in these services. Around 19% had not sought any such services since the pandemic began. In a subsequent question, 51% of respondents reported approaching and receiving services from OPDs and NGOs during the study period. Overall, the demand for services from government was low (37%), with civil society organisations (25%), private facilities and service providers (8%) and family and friends (20%) providing most of the needed assistance as other support mechanisms weakened or failed people with disabilities.

The data reported here – running out of money and being hungry – were new experiences for people with disabilities and would have increased pressure on the non-profit sector to provide support. One crucial challenge related to the government’s initial attempt to provide food for those in need was that the messages were either not reaching people with disabilities or they were unable to access the supply points. In another instance, 31% of respondents reported being unable to access public transport at times and turning towards OPDs for support.

Around 60% (*n* = 1112) of respondents reported the need for a professional carer or somebody to support them with daily living. Of this group, 73% (816) reported an interruption in this support because of COVID-19 and lockdown regulations. Approximately one-fifth (21%) of this group reported no interruption in caregiver support. When asked about how long carer support had been interrupted, 61% reported that this was a day, 10% reported a week, 18% reported interruptions of a couple of months. Worryingly, for 9% of the respondents these interruptions were continuing at the time of the survey. This might be because of a persistent fear of their carers catching the virus and transmitting this to respondents, most of whom had not been vaccinated at the time of the survey.

### Perspectives of government provision and responses

The authors asked respondents to indicate their perceptions about the state and others (NGOs) handling of the pandemic and support offered. Perceptions were the same for the government generally, for the government health and social sectors and for the NGO sector. Approximately 50% of the respondents felt that all three sectors were doing a bad job and around 30% felt they were doing a good job, while 10% were ambivalent about the performance of these three sectors.

**FIGURE 3:**
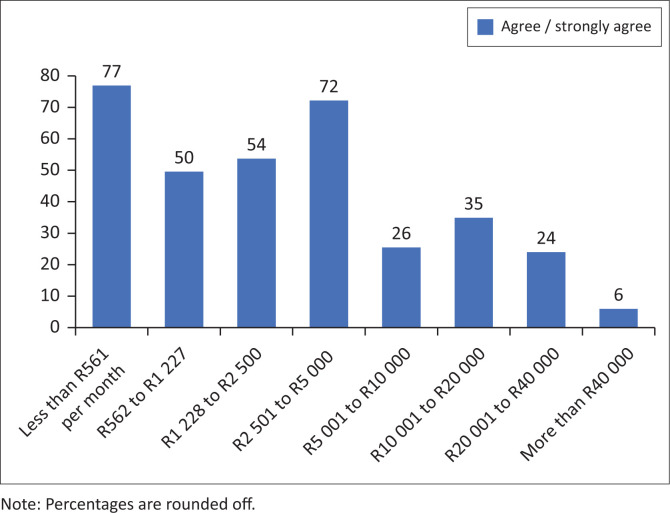
Government had taken the needs of persons with disabilities into account by income (*N* = 1857).

The authors asked whether respondents perceived that the government had adequately taken their specific needs into account during the pandemic. Somewhat surprisingly and seemingly in contradiction – given that over half had been very critical of the government generally and of the health and social sector – 55% reported that the government had taken the needs of persons with disabilities into account. On the other hand, 18% felt that this was not the case; 7% did not know and 20% reported uncertainty in response to this question. However, when the data are disaggregated by the respondents’ personal income, it shows that the higher their income the less likely the respondents are to agree or strongly agree that government had taken the needs of persons with disabilities into account (Figure 3). For example, 77% (*n* = 183) of those respondents earning less than R561.00 per month and 50% (*n* = 121) earning R562.00 to R1 227.00 per month thought government had taken the needs of persons with disabilities into account when compared with those who earn R5 001.00 to R10 000 (26%, *n* = 42) and between R10 001.00 and R20 000.00 (35%, *n* = 37). This difference may be a result of people with higher incomes expecting government to do more, but having less actual experience of using government services, while those with lower incomes and being more reliant on government for support and possibly more experienced in the type of services that are actually delivered in ‘normal’ times are more accepting of the limited interventions. Uncertainty is clarified in some of the qualitative responses, there being an impression that while government had put some interventions in place these were largely not known about and poorly communicated. Food relief and food parcels were indicated as an example.

Those disability-specific interventions that were known, included the increase in social grants for those already receiving disability and other social grants and the introduction of the social relief of distress grant (SRD) of R350.00 per month (a very small amount). Of those respondents receiving grants, 33% were getting the SRD, 31% received the disability grant, 10% the old age pension and 1% the care dependency grant for children with disabilities. At the age of 18 years the disability grant replaces the care dependency grant, and at the age of 60 years the pension replaces the disability grant, although the values are the same. Those receiving the SRD were not receiving any disability or old age grant indicating that a third of the respondents would not ‘normally’ receive a disability grant. Similarly, by receiving the SRD it is evident that these people were unemployed at the time. Of the respondents, 35% reported being unemployed and only 37% reported either self-employment, fulltime-, parttime- or casual employment at the start of lockdown in March 2020.

Respondents strongly supported the idea of disability-disaggregated monitoring during disasters and during this pandemic (83%), so that people are identified to receive assistance. Many felt the onus on addressing the disadvantages they experienced during the pandemic appears to have been on the under-resourced network of OPDs, who tend to support many of those with disabilities who fall outside of the government system. Many OPDs do not receive government funding on a regular basis but rely on the goodwill of private sector and foundations to deliver services. There was a general feeling that government could do more. It was observed that communication should be improved and accessibility features should be inherent in news broadcasts and other televised information about this and other disasters. While South African Sign Language (SASL) was available on television news broadcasts and has been for some time, some said they could not see the SASL interpreter and others needed captions as they did not understand SASL.

It was also found that the disaster regulations and other interventions were largely focused on the control of the virus and failed to recognise the negative and disruptive effects that the measures themselves would have on people with disabilities. The government failed initially to recognise the importance of regular disability-services for people with disabilities and shut these down during the first 2 months of the pandemic and only later reopened them. Of the approximately 150 disaster regulations promulgated up to July 2021, very few focused on persons with disabilities, and these had a strong emphasis on protection from the virus and the distribution of protective gear and equipment to safeguard health and social workers and teachers and learners (authors’ own reference). Such actions seem inadequate when considering the needs of the many people with disabilities who fall outside of the health and social system and would not be engaging with state employees or attending school. Given the speed with which government imposed a very harsh lockdown on all its citizens most people were unprepared, a situation that led to panic buying of commodities anticipated to be crucial during the forthcoming months. After the first 2–3 months transitions were rather rapid and fluctuated as South Africa entered different ‘waves’ of infection and levels of lockdown. More than 50% of respondents felt that level 3 and level 3-adjusted were the most acceptable levels because movement was not curtailed and necessary services were again available. The majority considered other levels to be either too severe or too relaxed.

### Demand for coronavirus disease 2019 vaccine

Slightly more than 80% of respondents knew of somebody who had been infected by the virus, of which 36% included the respondents themselves. When asked how likely they believed it was that they might get infected with COVID-19 in the forthcoming months, almost two-thirds (64%, *n* = 1192) of the respondents stated this was unlikely. On the other hand, just under a quarter (23%, *n* = 422) felt there was a chance of becoming infected. Other respondents were uncertain (11%). Most male (71%) and female (73%) respondents felt there was a little chance of infection in the forthcoming months. Despite a low fear of infection, 85% of the respondents indicated a strong keenness to be vaccinated, while only 7% were unwilling. Disaggregation by gender shows that a slightly greater share of male respondents (87%) than female respondents (81%) were willing to be vaccinated.

At the time of the survey, 5% of all respondents had been vaccinated. This small share of vaccinated respondents is because age cohort was the only criteria used to schedule the roll-out of the South African vaccination programme at the time. There was some objection by respondents to this approach. Qualitative responses illustrate that many objectors wanted the vaccination as a means of preventing infection in the context of feeling at increased risk. While respondents acknowledged that having an impairment did not make all or even most of them susceptible to infection with the coronavirus, there was a recurrent view in the qualitative data that people felt they needed it because they were at high risk of infection for a variety of reasons, not necessarily linked to clinical conditions. Some were more at risk because of specific and severe types of impairments, often because of immobility and related secondary conditions (e.g. respiratory vulnerabilities or being immunocompromised). However, others may have felt at risk for other non-clinical reasons related to their living situation or to their pre-existing disadvantage. For example, some respondents lived with and relied on people for their daily care and these people had become infected with the coronavirus. These respondents felt they were facing a greater risk than other people and wanted protection from the virus because of their reliance on those who were or had been infected. Please amend to

Interestingly, some respondents had managed to get vaccinated although they were not officially eligible on purely age criteria.

### Social and emotional impacts

Clearly most South Africans, whether living with disabilities or not have been negatively impacted, both emotionally and psychosocially, by COVID-19 and the intervention measures introduced to control the virus and prevent contagion. The situation for persons with disabilities is often exacerbated. In the first 17 months of the pandemic, respondents had a range of emotional experiences including anger, frustration, loneliness and boredom. A total of 60% reported feeling stressed, while 52% reported fear and 54% observed feeling depressed. Only 23% indicated feeling happy at any stage during the first 17 months. These reports are unsurprising. People with disabilities had lost jobs that are often difficult for them to get in ‘normal’ times and had experienced salary cuts, thus economic hardship had an emotional impact.

As reported here lockdown and mitigation interventions badly disrupted access to general needs, such as food, hygiene and electricity, as well as access to crucial rehabilitation, medication or healthcare services. Those reliant on assistive devices encountered challenges in keeping these in working order during the first two levels of lockdown when regulations closed relevant service providers. Such a situation is deeply depressing as these devices are necessary for daily functioning, communication and socialisation. Those living alone or those who depended on carers for daily living had these interrupted by needs to self-isolate, curfews, and associated travel restrictions. They subsequently experienced lengthened periods of loneliness.

Others depend on rehabilitative care and necessary medication and the inability to access these services because of their temporary closure or inability to access transport could result in the heightening of psychosocial and physical impairments. Such circumstances inevitably place great psychosocial and emotional toll on some people with disabilities who need these services to function daily and ensure a consistent level of independence and dignity.

## Discussion

Looking at the analysed data it is clear that the findings echo those from many other studies carried out globally, with different populations and using diverse methodologies as outlined here (Brennan et al. 2020; Christensen 2020; McKinney et al. 2021; Meaney-Davis 2020).

The prediction made by many authors and agencies at the start of the pandemic, that people with disabilities were at risk of exacerbated exclusion and disadvantage has sadly been shown to be prescient globally and in South Africa. This study is believed to be the largest looking at the experiences of people with disabilities in a single county, certainly in Africa but possibly globally. Other studies have focussed specifically on particular sectors such as health, education, employment or social protection and relief (Banks et al. 2021). Some have looked at specific subpopulations, for example, by impairment, gender or age. The authors considered a variety of options to collect data on a wide spectrum of topics and a broad range of people.

The questions about economic impacts, service interruption, vaccine access and people’s perspectives of government intervention all show predominantly negative impacts and that notable proportions of people who had previously not experienced such concerns, experienced unusual and upsetting events such as not being able to get their usual care/assistance, disability specific services or being hungry. It does seem (unsurprisingly) that people’s perceptions are somewhat related to their own financial status and their expectations and previous experience of services and may also be related to the type and severity of their impairments, although it has not been analysed for this article. Experience of change of circumstances and the extraordinary situation of a pandemic was new for everyone, and thus it is perhaps predictable that people will have an array of ideas about what government could or should do in response. Those with more awareness of human rights statutes and rhetoric might be more critical and have a stronger sense of entitlement to equal provision. Others may be rather resigned to inequality and have internalised the oppression they often experience. This might explain the sometimes contradictory responses to questions about the adequacy of government responses. There is a strong sense of being left out or forgotten and that the service providers, whether government or others do not consider their needs specifically enough.

There is a perceived need for prioritisation for vaccinations and there is awareness of increased clinical risk of catching the virus for some with underlying health conditions causing or arising from having an impairment and having a weakened immune system. Impairment specific concerns emerge relatively rarely, but this might be because the survey did not probe on these in depth. However, it is observed that people with certain impairments found it challenging to access information and updates and communal service points and other facilities where support was delivered. Although sign language supported information is available on TV, there is a need for this to be more widespread and for other inclusive, adapted formats.

Significant numbers are seemingly overlooked by primary grants, while a share of this population reported high levels of food insecurity and hunger, employment challenges and high levels of worry and stress. Although they are included in general anti-discriminatory legislation this has not protected them specifically enough during the pandemic. Specific rights as indicated in the Bill of Rights appear to have been ignored. It is observed that the right to dignity, as indicated in section 10 of the Constitution, has been overlooked. During the early months of lockdown, some respondents were unable to receive rehabilitative treatment or get their assistive devices serviced or repaired. Emergency regulations prevented carers from going to their clients, leaving them alone without assistance. Furthermore, basic rights to access healthcare facilities and services, sufficient food and water, and social security were denied, with a large share, including other household members, going to bed hungry. This neglect of the specific needs for disability rights to be intentionally upheld continues a pre-COVID-19 feeling of exclusion and loss of dignity.

People with disabilities reported isolation from friends and family. The barriers they encounter in accessing information and health services were intensified during the pandemic as they were not seen as a priority for access to health and other services (McKinney et al. 2020) in contrast to other groups such as the elderly, as was evident in the vaccine programme scheduling criteria. Having to shift from reliance on professional or outside care, to dependency on family, friends and OPD personnel as they attempted to address the gaps and secure needed goods and services seems to have placed additional pressure on all.

Reflecting on the authors’ choice of approach and methodology and given the restricted range of options available because of the COVID-19 regulations, they feel that they have achieved their aim of gathering a sizeable and demographically inclusive set of data about people with disabilities’ experiences of COVID-19, lockdowns and their implications in South Africa. The decision to run the study as a partnership between two academic research institutions with different disciplinary strengths, alongside a national umbrella organisation has proved to be a good one. The collaboration between the three organisations produced thinking and working that was more than the sum of its parts. To a large extent the authors were not only in agreement about some basic principles (e.g. about the need and purpose of the study, disability-related language, inclusive practice etc.) but also found that in discussions at various points in the process, they learnt more and interesting things about their respective perspectives and priorities. For example, the NCPD contributed vital knowledge of the range of living situations of people and about OPDs and their networks. The IDS and HSRC understand the complexity of trying to reach a dispersed and diverse population to complete the survey across the country. Reciprocally, the two research organisations specifically contributed an international perspective on disability inclusive research and skills in storing and analysing quantitative data, respectively, (IDS and HSRC), as well as having joint understandings about the nature of research and an interest in practical and action-orientated explorations with explicit links into policy formation. This was a new collaboration and a very effective one. Joint dissemination activities were productive and enjoyable. Given more time the authors would have liked to spend more time in exchange of ideas between the three partners, performed more joint analysis and writing and have had more direct contact with some of the respondents to validate their analysis.

### Limitations of the study

While the authors believe this is the largest and most comprehensive known study to explore the experiences of people with disabilities in a single country worldwide, several limitations were identified, which should be recognised when interpreting the results. The scheduling of the survey may have had an impact on its results. The survey ran between July and August 2021, and at this time, South Africa experienced violent protests and sociopolitical unrest (Vhumbunu 2021). This extraordinary period may have influenced who was able to participate and how they responded. People may not have been able to purchase data. Some real threats to their lives might have affected their willingness to respond to the survey at all. Emotional and psychological questions may have been viewed negatively. Completing a survey would not be a priority in such circumstances. Also, the COVID-19 regulations themselves in place at the time of the survey may have had an impact on response rates as support would have been interrupted.

The methodology required access to and familiarity with online technology and connectivity to participate. Despite every effort to make the survey accessible, it is possible that certain groups (e.g. people with visual or cognitive impairments) may have found it harder to participate. Also, participants with little literacy or who are very poor may have found the online nature of the survey to be a barrier. It is likely that more technologically literate potential respondents felt more able to participate. This is suggested by the education profile of participants which is higher than that of the general population and the proportion of relatively young people (18–40 years old). Participants with higher education levels may also have found it easier than the general population to access information about COVID-19, so their responses about this may be more positive than for other people. In addition, the spread of respondents across the country was uneven, so although all South African provinces are represented, the majority of respondents are from Gauteng (63.8%). Also important to note, the survey was largely quantitative in nature, providing limited access to knowledge about the contextual living conditions of the respondents. A further constraint is that the survey was only in English and this may have excluded some people. Further qualitative and participatory inquiry may address such knowledge gaps. Finally, in this study there is no indication about how many people received the survey but chose not to respond and what the reasons for this were.

## Conclusion

Predictions were made at the start of the COVID-19 outbreak that people with disabilities globally would be at risk of exclusion from relief and mitigation services and would also experience an increase in the many disadvantages they so often experience in ‘normal’ times. There was a fear that the substantial progress in improving lives of people with disabilities that has been made in recent years, such as increased recognition of the equal rights of people with disabilities, recognition as citizens, adequate access to services within mainstream provision (e.g. in health, rehabilitation, education, employment) would be reversed. Hard won victories in relation to these and other aspects would then have to be fought for again.

However, on the positive side perhaps, the evidence from our study and others may raise awareness of these negative consequences and prompt more disability inclusive policies and practices globally (Humanitarian Disability Charter 2022; IASC 2019). Across contexts one should be able to make the case for new and revised anticipatory policies and practices, which have disability inclusion built in as standard from the start, across sectors (Wickenden et al. 2021). Specifically in South Africa, if a new framework on responses to disasters and crises is being formulated, the need for which has been recognised by relevant ministers, then there is now no excuse for ignoring the evidence that people with disabilities have suffered more than most from the direct and indirect consequences of the pandemic. Better outcomes in future can only be achieved with improved disability inclusive planning and specific support for people with disabilities in times of crisis.

Lastly, the key recommendations, which emerged from the data are summarised as follows:

- Inclusive disaster management and mitigation planning processes are required. Government must immediately address the gaps that currently exist in the disaster and risk framework for people with disabilities to ensure that they are explicitly included in the discussions about pandemic mitigation and the recovery processes and that their specific needs are catered for in legislation and regulations. Collaboration between state and Organisations of People with Disabilities (OPD) is essential to ensure that plans are always disability inclusive.
- Inclusive baseline data collection: Data on people with disabilities is fragmented, preventing comprehensive mapping and tracing that could enable disability-inclusive interventions.
- Cross-sectoral inclusive service provision: Interventions during crises must ensure accessibility to necessary rehabilitation, care, health services, food and nutrition security, and that access to hygiene measures is not interrupted for people with disability.
- The psychosocial support: A calculated effort must be made to address the negative impacts of stress, isolation, interrupted therapy, carer, rehabilitative and mental health services and economic challenges that the pandemic has placed on people with disabilities.
- Communication challenges: There is a need for improved means of communication with people with disabilities about the nature of the crisis, regulations and provisions available. Some found it challenging to communicate with staff at communal service points and other facilities where support was delivered. Others, those with sensory impairments, struggled to obtain information and updates via television. While sign language supported information is available on TV, there is a need for more of this and other inclusive, adapted formats for people with specific needs. For example, many speeches are prepared in advance and could easily be delivered with closed captions and with voiceover for those who do not understand English or sign.

Finally, it is interesting to notice that these recommendations arising from our study, mirror very closely those of many other studies and agencies globally. This is in a way encouraging as we should be able to lobby with a united voice at different levels of power, influence and action to improve disability inclusive responses before the next emergent crisis.
